# Upgrading Mixed Plastic Waste through Industrial Symbiosis: Pseudoductile Regenerated Cellulose Fiber-Reinforced Shredder Residue Composites

**DOI:** 10.1021/acsapm.4c02728

**Published:** 2024-11-18

**Authors:** Kanjanawadee Singkronart, Jiayi Amy Sun, Siti Ros Shamsuddin, Koon-Yang Lee

**Affiliations:** † National Metal and Materials Technology Centre, National Science and Technology Development Agency, 10210 Pathum Thani, Thailand; ‡ Department of Aeronautics, Imperial College London, South Kensington Campus, SW7 2AZ London, United Kingdom; § Institute for Molecular Science and Engineering (IMSE), Imperial College London, SW7 2AZ London, United Kingdom

**Keywords:** recycling, plastic waste, Rayon fiber, waste reinforced waste composite, wet powder impregnation

## Abstract

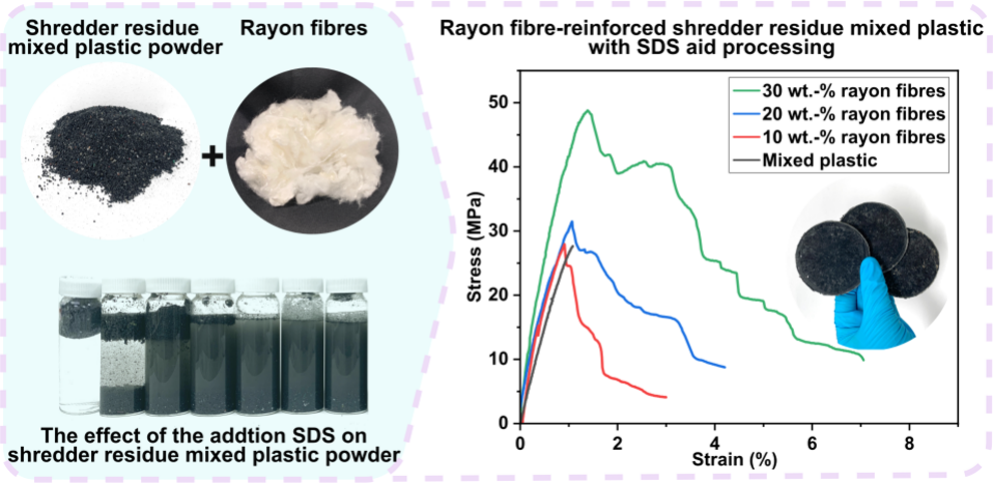

The mechanical performance of mixed plastic waste from shredder residue is hindered by brittleness and catastrophic failure, limiting its potential applications. In this study, the mechanical properties of mixed plastic is enhanced by reinforcement with rayon fibers through a wet powder impregnation process to leverage the fiber's ductility and entanglement. However, mixed plastic remains poorly dispersed in water during the composite manufacturing, resulting in poorly consolidated composite, which further deteriorates the mechanical properties of mixed plastic from 1.5% strain-at-break to 0.7%. To address this issue, the addition of sodium dodecyl sulfate (SDS) surfactant is explored, where the optimal concentration is found beyond the critical micelle concentration at 10 mM. Lowering the surface tension of water and the adsorption of the SDS on the mixed plastic powder surface facilitated homogeneous dispersion of mixed plastic particles, resulting in well-consolidated rayon fiber-reinforced composites. The 30 wt % rayon fiber-reinforced mixed plastic composite prepared with SDS demonstrated a progressive failure behavior, exhibiting a strain-at-break of 8% and a remarkable 350% increase in impact strength compared to unreinforced mixed plastic. This approach provides a platform to overcome the inherent limitations of mixed plastic waste, offering waste-derived plastic alternatives and reducing the need for fossil-derived virgin materials for a wide range of noncritical applications.

## 1 Introduction

In our fast-paced and technology-driven world, there has been a significant increase in the proliferation of electrical and electronic equipment (EEE), as well as motor vehicles. According to the European Union (EU), 12.5 million tonnes of EEE^1^ and 405 million tonnes of motor vehicles^2^ were put on the market in 2020. In 2023, these numbers have increased by 5.5 and 3% for EEE and motor vehicles, respectively.^3,4^ This also has the unfortunate consequence of increasing the amount of waste electrical and electronic equipment (WEEE), as well as end-of-life vehicles (ELV). It has been estimated that more than 10 million tonnes of EEE and motor vehicles are scrapped every year.^1^

In the scrapping process, fluids and batteries are first removed, followed by indifferent shredding to prioritize the recovery of the metallic fraction,^5,6^ as it has a higher economic value than the nonmetallic fraction. The remaining nonmetallic waste, also known as the shredder residue, is typically composed of plastics (ca. 40% of ELV^5^ and ca. 15% of WEEE^7,8^ end up as shredder residue). Due to its low economic value, there is no incentive to sort the different materials from one another, and the typical end-of-life options for shredder residue are landfill or incineration.^9,10^ Plastics landfilling can result in the leaching of hazardous chemicals, such as brominated flame retardants, and the releasing of microplastics into soil and water systems.^8,11,12^ The incineration of plastics and other nonmetallic materials can produce toxic byproducts, such as polycyclic aromatic hydrocarbons, dioxins, or furans, and release particulate matter into the atmosphere.^12^ It is estimated that 100,000 tonnes of particulate matter less than 10 μm in size (PM_10_) are emitted annually in the EU alone.^13^

A simple and pragmatic solution to reduce the environmental footprint of shredder residue mixed plastic is to repurpose them directly as it is.^14,15^ However, this will result in a brittle polymeric product due to the immiscibility of the different polymers in the highly heterogeneous shredder residue mixed plastic.^16−18^ It had been previously shown that an immiscible polymer blend consisting of shredder residue mixed plastic (acrylonitrile butadiene styrene, polystyrene, polypropylene, and polyethylene) possessed a tensile modulus of 2.9 GPa and a tensile strength of 32 MPa.^19,20^ While these values are not significantly different from the tensile properties of virgin ABS, PS, and PP, the strain-at-failure of this mixed plastic was found to be only 1.5%, which limits its potential in a variety of engineering applications. As a comparison, virgin ABS, PS, and PP have a strain-at-failure of 77, 5, and 578%, respectively.

Cellulosic fibers have garnered significant attention recently as reinforcement for polymers due to their renewability, nonabrasive nature, low cost, high specific stiffness and strength, as well as abundance in supply.^21,22^ In this context, regenerated cellulose fibers have the potential to enhance the poor mechanical performance of an immiscible blend of shredder residue mixed plastic. The tensile modulus and strength of regenerated cellulose fibers have been measured to be 12–42 GPa and 400–600 MPa, respectively.^23,24^ More importantly, regenerated cellulose fibers possess a strain-at-break of around 10–24%,^24−25,28^ which could enhance the ductility and toughness of an immiscible polymer blend derived from shredder residue mixed plastic. In addition to this, the fast fashion sector generates a substantial amount of both preconsumer (off-cuts) and postconsumer textile waste (incl. regenerated cellulose fibers), which take up 5% of space in landfill.^29^ Combining waste from the WEEE and ELV sectors with those from the fast fashion sector offers the possibility of repurposing different types of waste into higher value products, achieving industrial symbiosis.^30^

Several studies have been conducted to investigate the mechanical performance of model regenerated cellulose fiber-reinforced thermoplastics. Ganster and Fink^31^ manufactured randomly oriented short viscose rayon fiber-reinforced poly(lactic acid) (PLA) composites. At a fiber loading of 25 wt %, a tensile modulus and strength of 4.5 GPa and 110 MPa, respectively, have been obtained. By comparison, neat PLA has only a tensile modulus of 3 GPa and a tensile strength of 70 MPa. More importantly, the impact toughness, measured using Charpy impact test, increased from 40 to 70 kJ m^–2^. Similar observations in terms of improved ductility and toughness with regenerated cellulose reinforcement were also obtained by Bax and Müssig^32^ for randomly oriented short Cordenka fiber-reinforced PLA composites, Graupner and Müssig^33^ for randomly oriented short Lyocell fiber-reinforced polypropylene (PP) and PLA composites, as well as Shamsuddin et al.^21^ for continuous Cordenka fiber-reinforced polyhydoxybutyrate (PHB) composites. Inspired by these studies, this work reports the use of ductile regenerated viscose rayon fiber as reinforcement for brittle shredder residue mixed plastic to improve its mechanical properties. The processing of shredder residue mixed plastic with staple rayon fibers and sodium dodecyl sulfate (SDS) as the processing aid is discussed. The internal structure, tensile properties, work of fracture, as well as impact properties of the model rayon fiber-reinforced shredder residue mixed plastic composites are also investigated and reported.

## 2 Experimental Section

### 2.1 Materials

Industrial shredder residue mixed plastic granules (40–50% acrylonitrile butadiene styrene (ABS), 30–40% polystyrene (PS), 10–15% polypropylene (PP), 2% polyethylene (PE) and 5% rubber and others) were obtained from Axion Polymers (Manchester, U.K.). Prior to use, the shredder residue mixed plastic granules were melt-processed at 210 °C in a corotating twin-screw extruder (Eurolab XL, screw diameter = 16 mm, L/D = 25, Thermo Fischer Scientific, Karlsruhe, Germany) and pelletized (Haake VariCut, Thermo Fischer Scientific, Karlsruhe, Germany) into 1 mm long pellets. They were then cryogenically ground (Blitz 5687, SQ Professional Ltd., Enfield, U.K.) for 5 min into a powder form. The average particle size (*d*_50_) of this powder was 200 μm. The particle size distribution of this powder can be found in Figure S1 of the Supporting Information. Staple viscose rayon fibers (filament diameter = 12 ± 2.5 μm, length = 3.5 ± 1.2 cm, density = 1.53 g cm^–3^) off-cuts were kindly supplied by a commercial retailer. Sodium dodecyl sulfate (SDS) (ReagentPlus, purity ≥98.5%) was purchased from Sigma-Aldrich (Dorset, U.K.) and used as a processing aid in the composite fabrication.

### 2.2 Manufacturing of Model Rayon Fiber-Reinforced Shredder Residue Mixed Plastic Composites

**Figure 1:**
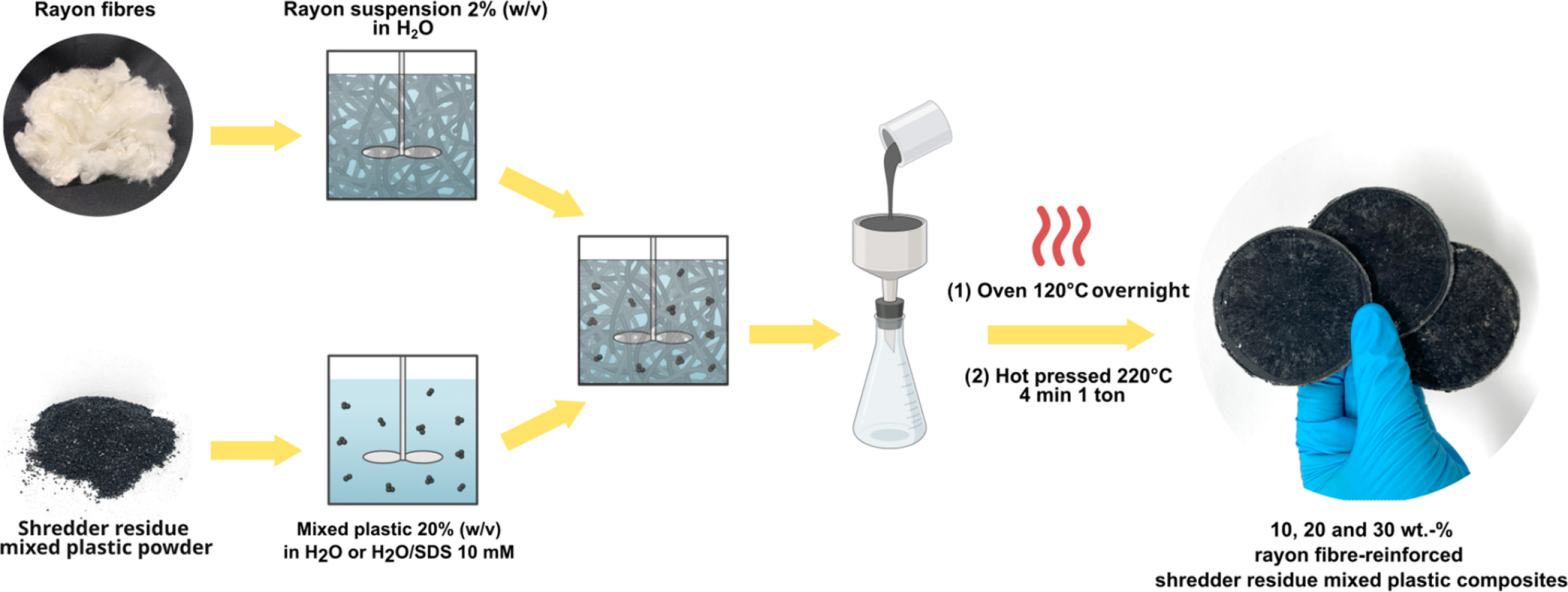
Schematic diagram summarizing the manufacturing process of rayon fiber-reinforced shredder residue mixed plastic composites.

Wet powder impregnation is a well-known composite manufacturing technique to produce continuous fiber-reinforced thermoplastic tapes or prepregs.^21,34−37^ Typically, a continuous fiber tow is pulled through a polymer powder suspension, picking up the polymer powder through capillary actions and interlocking them between the fibers. The impregnated fiber tow is then subjected to heat treatment to evaporate the liquid, followed by melt-consolidation into unidirectional fiber-reinforced thermoplastic tapes. This wet powder impregnation technique was used to manufacture shredder residue mixed plastic composites reinforced with 10, 20, and 30 wt % staple rayon fibers. The composite fabrication process is presented schematically in Figure 1. First, 2 (w/v)% staple rayon fibers in water suspension was prepared and stirred at 20 rpm for 15 min using a blade stirrer (Hei-Torque Expert 100, Heidolph Instruments GmbH & Co., Schwalbach, Germany) prior to leaving them to soak overnight. Separately, the previously processed shredder residue mixed plastic powder was dispersed at a consistency of 20 (w/v)% in either pure water or an aqueous SDS solution under magnetic stirring at 1000 rpm for 15 min. An SDS concentration of 10 mM (ca. 0.3 wt %) was chosen based on a parametric study conducted (see Section 3.1). The staple rayon fiber in water suspension was then poured into the suspension containing the shredder residue mixed plastic powder. For samples prepared with SDS as the processing aid, the concentration of SDS in the resulting rayon fibers/shredder residue mixed plastic powder suspension was adjusted to 10 mM through the addition of more SDS. The rayon fibers/shredder residue mixed plastic powder suspension was then stirred magnetically for another 15 min prior to vacuum filtering onto a 55 mm diameter filter paper (Grade Whatman 1 cellulose filter paper, 11 μm particle retention, Cytiva CTH Holding Ltd., Buckinghamshire, U.K.). The filter cake was carefully removed and sandwiched between fresh sheets of filter paper placed between sheets of blotting paper (Grade 3MMCHR, GE Healthcare, Buckinghamshire, U.K.) and press-dried under a weight of 4 kg at 120 °C overnight. The dried filter cake was placed between two Mylar films (MY125, Lohmann Technologies UK Ltd., Milton Keynes, U.K.) and ramped to 220 °C at a rate of 10 °C min^–1^, followed by hot pressing (Model 4122CE, Carver Inc., Wabash, IN) for 4 min under a weight of 1 tonne to produce the model rayon fiber-reinforced shredder residue mixed plastic composite. A hold time of 4 min was chosen, as longer exposure time will lead to the discoloration of the rayon fibers.

### 2.3 Processing of Shredder Residue Mixed Plastic Powder

As a benchmark, the shredder residue mixed plastic powder was also processed into dog bone-shaped and rectangular test specimens using injection molding (Haake Minijet Pro Thermo Fischer Scientific, Karlsruhe, Germany). The barrel and mold temperatures of the injection molder were set to be 210 and 40 °C, respectively. The injection pressure and time were set to be 65 MPa and 10 s, respectively, followed by a holding pressure and a time of 65 MPa and 60 s, respectively. The injection molded test specimen possessed an overall length of 65 mm, a thickness of 3 mm, and a gauge length of 10 mm, and the narrowest part of the dog bone specimen was also 3 mm. The rectangular test specimen possessed an overall length of 80 mm, a width of 13 mm, and a thickness of 3 mm.

### 2.4 Material Characterizations

#### 2.4.1 Surface Tension of Aqueous SDS Solutions

As part of the investigation to identify the optimum SDS concentration to disperse the shredder residue mixed plastic powder in water, the surface tension of the various aqueous SDS solutions was determined. A pendant drop was formed from a blunt tipped stainless steel needle (Hamilton 91017-G17, Fisher Scientific UK Ltd., Leicestershire, U.K.) with an inner diameter of 1 mm and photographed using EasyDrop (Krüss ADVANCE, version 1.9.0.8, Krüss GmbH, Hamburg, Germany). The surface tension value was then obtained by fitting the shape of the pendant drop formed with the Young-Laplace equation. An average of 20 measurements is presented for each SDS concentration.

#### 2.4.2 Dispersion of Shredder Residue Mixed Plastic Powder in Aqueous SDS Solutions

The quality of the mixed plastic powder in a water dispersion at different SDS concentrations was evaluated visually. Suspensions of 20 (w/v)% shredder residue mixed plastic powder were prepared under magnetic stirring at 1000 rpm for 15 min. The stability of the mixed plastic powder in the respective suspensions was recorded at various time points (i.e., 10, 50, 100, and 1000 s) after magnetic stirring was stopped.

#### 2.4.3 Static Water/Air Contact Angle Measurements

Static water–air contact angle measurements were performed using Krüss EasyDrop (Krüss GmbH, Hamburg, Germany). Two different substrates were investigated: a lightly compacted bed of shredder residue mixed plastic powder to obtain the apparent contact angle (θ*) of the mixed plastic powder and a uniform film of melt-consolidated shredder residue mixed plastic powder to obtain the equilibrium contact angle (θ^E^) of the mixed plastic matrix. For θ* measurement, the shredder residue mixed plastic powder was compacted under a pressure of 50 kPa in a rectangular die (4.40 cm × 1.75 cm) and affixed onto a glass slide based on a previously described protocol.^38^ For θ^E^ measurement, the shredder residue mixed plastic powder was hot-pressed at 220 °C under a weight of 2 tons into a film of ∼0.7 mm in thickness before affixing it on a glass slide using double-sided tape. A water droplet with a volume of 10 μL was carefully placed on the substrate, and the water/air contact angle formed was analyzed using ellipse fitting conducted on Krüss ADVANCE (version 1.9.0.8, Krüss GmbH, Hamburg, Germany). An average of 20 droplets is reported for each substrate.

#### 2.4.4 Morphology of Model (Rayon Fiber-Reinforced) Shredder Residue Mixed Plastic(s)

Scanning electron microscopy (SEM) was used to investigate the morphology of the shredder residue mixed plastic powder, as well as the tensile fracture surface of the model rayon fiber-reinforced composite. It was performed using a large chamber electron microscope (S-3700 N, Hitachi, Tokyo, Japan). An accelerating voltage of 15 kV was used. Prior to SEM, the samples were mounted onto aluminum stubs using carbon tabs. It was then Au coated (Agar Auto Sputter coater, Agar Scientific Ltd., U.K.) using a coating current of 40 mA for 20 s.

#### 2.4.5 Porosity of Model (Rayon Fiber-Reinforced) Shredder Residue Mixed Plastic(s)

He pycnometry (Accupyc II 1340, Micromeritics Ltd., Hexton, U.K.) was used to determine the true density of the staple viscose rayon fibers (ρ_f_) and the shredder residue mixed plastic powder (ρ_m_). The envelope density (ρ_e_) of the (fiber-reinforced) shredder residue mixed plastic(s) was calculated by dividing the mass of the sample by its envelope volume. With the weight fraction of the reinforcing fibers (*W*_f_) known, the void-free density of the (fiber-reinforced) shredder residue mixed plastic(s) (ρ) was computed using
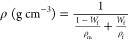
1The porosity (*P*) of the (fiber-reinforced) shredder residue mixed plastic(s) was then calculated using the equation:
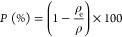
2An average of five specimens is reported for each sample.

#### 2.4.6 Tensile Properties of Model (Rayon Fiber-Reinforced) Shredder Residue Mixed Plastic(s)

Tensile properties were investigated in accordance with ASTM D638-14. The test was conducted using an Instron universal tester (Model 5960, Instron Corporation, High Wycombe, U.K.) equipped with a 10 kN load cell. A total of five specimens were tested for each sample. Prior to tensile testing, the sample was cut into rectangular test specimens (50 mm × 10 mm × 3 mm) using a CNC router machine (Infinite 4006, AXYZ Automation Inc., Telford, U.K.), and a dotted pattern was stamped (IMT-ACC001, iMetrum Ltd., Bristol, U.K.) onto the surface of each test specimen. Glass fiber-reinforced epoxy end tabs (10 mm × 10 mm) were glued onto the test specimen using two-part cold curing epoxy resin (Araldite 2011, Huntsman International Ltd., Wiltshire, U.K.). This was to avoid the clamps of the tensile tester from damaging the test specimen, potentially leading to an earlier onset failure within the gripping zone. The exposed length of the rectangular test specimens was 30 mm. The strain experienced by the test specimen under uniaxial tensile loading was evaluated by monitoring the movement of these dots using a noncontact optical extensometer (IMT-CAM027, iMetrum Ltd., Bristol, U.K.). A crosshead displacement speed of 1 mm min^–1^, which corresponded to a strain rate of 0.06% s^–1^, was used in this characterization.

#### 2.4.7 Charpy Impact Strength of Model (Rayon Fiber-Reinforced) Shredder Residue Mixed Plastic(s)

Unnotched flatwise Charpy impact strength was characterized in accordance with BS EN ISO 179-1:2023 using a Charpy impact tester (Model 5102.100, Zwick/Roell Ltd., Herefordshire, U.K.). A type III specimen with dimensions of 40 × 10 × 3 mm was used. The swinging pendulum possessed an energy of 2 J, and the frictional loss in the measurement was estimated to be 10 mJ. At the point of impact, the velocity of the impactor was estimated to be 2.93 m s^–1^. The support span length used in the test was 24 mm. A total of five specimens were tested for each sample, and the average values are reported.

## 3 Results and Discussion

### 3.1 Dispersion Quality of Shredder Residue Mixed Plastic Powder in Water as a Function of SDS Concentration

**Figure 2:**
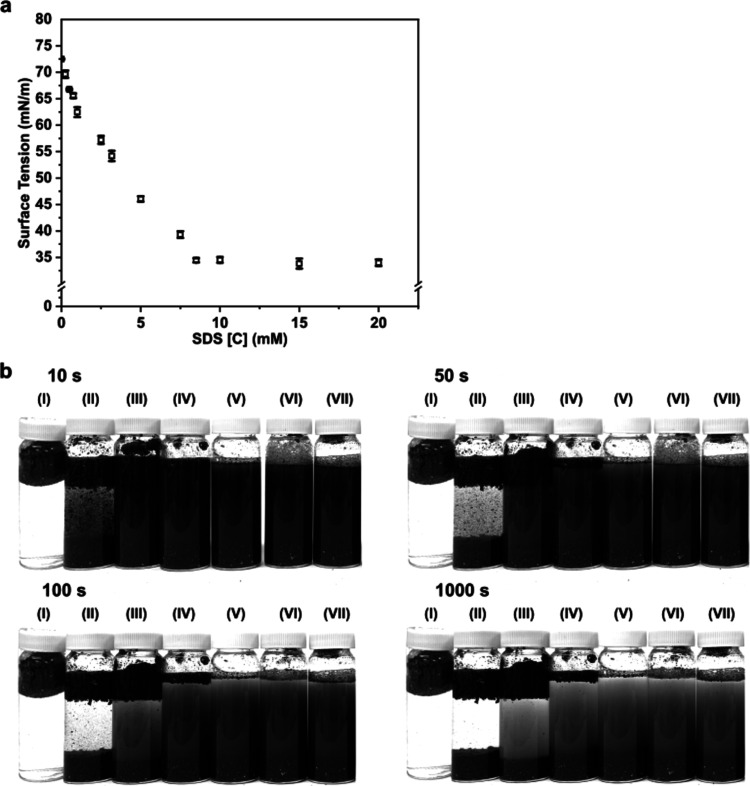
(a) Effect of SDS on the surface tension of water and (b) photographs showing the stability of 20 (w/v)% shredder residue mixed plastic powder dispersion at different periods of time in (I) pure water, (II) 2.5 mM, (III) 5 mM, (IV) 7.5 mM, (V) 10 mM, (VI) 15 mM, and (VII) 20 mM aqueous SDS solutions.

One of the most important aspects of the wet powder impregnation technique is the quality of the thermoplastic powder in water dispersion. As most engineering polymers are hydrophobic, surfactants are used. The thermoplastic-surfactant combination that had been studied include polyhydroxybutyrate (PHB)-Cremophor,^21^ polyetheretherketone (PEEK)-Cremphor,^37^ polyphenylenesulfide (PPS)-Cremphor,^39^ PEEK-Triton X100,^35,36^ and PEEK-Aerosol OT.^35^ Here, in this study, SDS was used as the surfactant to disperse the shredder residue mixed plastic powder in water. SDS is one of the highly utilized surfactants across diverse applications owing to its water solubility and remarkable efficiency in reducing surface tension.^40^ The reduction in the surface tension of water as a function of SDS concentration is presented in Figure 2a. The critical micellar concentration (CMC) of SDS in water, where the surface tension of the solution plateaued, was found to be ∼8 mM. This is consistent with those reported in the literature.^41,42^

**Figure 3:**
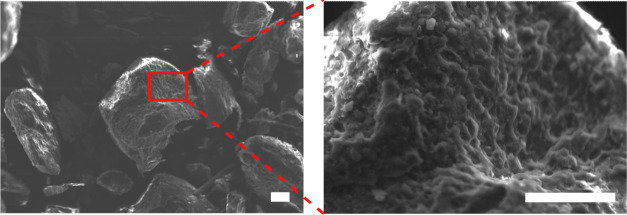
SEM images of the surface of the shredder residue mixed plastic powder. Scale bar = 50 μm.

The dispersion of the shredder residue mixed plastic powder in water at different SDS concentrations is shown in Figure 2b. Without SDS, the shredder residue mixed plastic powder stayed afloat. This stems from the hydrophobicity of the constituents in the mixed plastic. The water-in-air contact angle conducted on flat and smooth ABS, PS, PE, and PP films were reported to be 81,^43^ 92,^44^ 92,^45^ and 105°,^46^ respectively. The poor wettability of the shredder residue mixed plastic powder is further exaggerated by surface roughness resulting from the polymer grinding process (Figure 3). This exposed the heterogeneous microstructure of multicomponent polymer, which is the characteristic of immiscibility.^19^ We obtained a θ* for the shredder residue mixed plastic powder of 123 ± 3°, higher than those measured on a flat and smooth surface. This implies the formation of the Wenzel and Cassie–Baxter wetting states.^47−49^ In the Wenzel wetting state,^49^ if a surface is inherently hydrophobic (which it is in our case), roughness increases effective surface area and hence, the apparent contact angle following the equation cos θ* = *R* cos θ^E^, where  and is greater than unity. In the Cassie–Baxter wetting state,^48^ a composite surface between air and the solid material is formed, leading to an increase in apparent contact angle following cos θ* = ⌀_s_(cos θ^E^ + 1) – 1, where ⌀_s_ is the fraction of the solid/liquid interface on the material surface beneath the liquid.

Figure 2b also shows that with increasing concentration of SDS, the dispersibility of the hydrophobic shredder residue mixed plastic powder in the water phase improved. This can be attributed to the adsorption of SDS molecules onto the surface of the mixed plastic powder in the tail-down configuration, with the hydrophilic head exposed to the water phase.^50^ Note that the dispersion eventually sedimented once they are dispersed into the water phase as the shredder residue mixed plastic powder possessed a higher density than the surrounding water (Table 1). As the CMC was found to be ∼8 mM and a 10 mM aqueous SDS solution was found to be sufficient to fully disperse the shredder residue mixed plastic powder in water (see Figure 2b, bottle V), subsequent model rayon fiber-reinforced shredder residue mixed plastic composite manufacturing is based on this SDS concentration and compared with those produced without using SDS as the processing aid. It should be mentioned that SDS will also adsorb onto cellulose fibrils^51,52^ during the wet powder impregnation step, most likely with a head-down configuration due to the hydrophilicity of the rayon fibers.^51,53^

**Table 1:** Envelope Density (ρ_e_), Void-Free Density (ρ), and Porosity (*P*) of Shredder Residue Mixed Plastic and Rayon Fiber-Reinforced Shredder Residue Mixed Plastic Composites

sample	ρ (g cm^–3^)	ρ_e_ (g cm^–3^)		*P* (%)	
0 wt %	1.18 ± 0.01	1.16 ± 0.01		2 ± 1	
		with SDS	no SDS	with SDS	no SDS
10 wt %	1.20 ± 0.01	1.10 ± 0.02	1.00 ± 0.03	9 ± 2	17 ± 3
20 wt %	1.23 ± 0.01	1.07 ± 0.02	0.92 ± 0.04	13 ± 2	25 ± 4
30 wt %	1.26 ± 0.01	1.08 ± 0.03	0.80 ± 0.04	15 ± 3	35 ± 4

### 3.2 Porosity and Morphology of Model Rayon Fiber-Reinforced Shredder Residue Mixed Plastic Composites

Table 1 summarizes the porosity of the rayon fiber-reinforced shredder residue mixed plastic composites produced with and without using SDS as a processing aid during the wet powder impregnation step. The porosity of unreinforced shredder residue mixed plastic processed at 210 °C was found to be ∼2%. If the shredder residue mixed plastic granules were processed at a temperature less than 210 °C, poor consolidation will be observed based on our previous study.^19^ If a higher temperature was used, volatiles from the thermal degradation of rubber and other additives would be generated and trapped within the material, increasing its porosity. A trend could also be observed for the porosity of rayon fiber-reinforced shredder residue mixed plastic composites. Increasing the loading of rayon fibers led to an increase in the porosity of the resulting rayon fiber composites. Furthermore, composites produced using SDS as the processing aid possessed lower porosity than those produced without using SDS. At 30 wt % rayon fiber loading, the porosity of the rayon fiber-reinforced mixed plastic composite produced with the aid of SDS in the wet powder impregnation step was 15%. Without SDS, the porosity of the resulting composite was found to be 35%.

**Figure 4:**
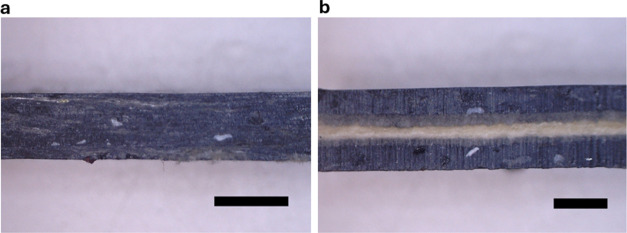
Exemplary microscope images of 30 wt % rayon fiber-reinforced shredder residue mixed plastic composites (a) with SDS and (b) without SDS as the processing aid. Scale bar = 2 mm.

To elucidate this further, the cross section of a model 30 wt % rayon fiber-reinforced shredder residue mixed plastic composite processed with and without using SDS were investigated (Figure 4). Microscopy images of 10 and 20 wt % rayon fiber-reinforced shredder residue mixed plastic composites processed with and without SDS as the processing aid can be found in Figure S2 of the Supporting Information. The rayon fibers are distributed within the shredder residue mixed plastic matrix when SDS was used (see Figure 4a), but without SDS, a laminated construct consisting of a layer of dried nonwoven rayon fibers sandwiched between two shredder residue mixed plastic rich matrix can be seen (see Figure 4b). Upon the mode I opening of the laminated construct, the rayon fibers at the midplane were found to be dry without any presence of the mixed plastic matrix (see Figure S3 of the Supporting Information). These results signify the importance of obtaining a homogeneous dispersion of shredder residue mixed plastic powder in water prior to composite consolidation. Rayon fibers (or cellulose fibers in general) are hygroscopic and will disperse in water during processing.^54^ The shredder residue mixed plastic powder phase, on the other hand, separates from the water phase (Figure 2b, bottle I). This led to the incomplete wetting between rayon fibers and the shredder residue mixed plastic matrix during processing. With the right concentration of SDS, the shredder residue mixed plastic powder can uniformly disperse in water (see Figure 2b, bottle V) and form a more homogeneous distribution of the polymer powder in the rayon fibers during the wet powder impregnation step. The difference in the architecture of the rayon fiber-reinforced shredder residue mixed plastic composites, ultimately, influences their mechanical properties.

### 3.3 Tensile Properties of Model Rayon Fiber-Reinforced Shredder Residue Mixed Plastic Composites

**Figure 5:**
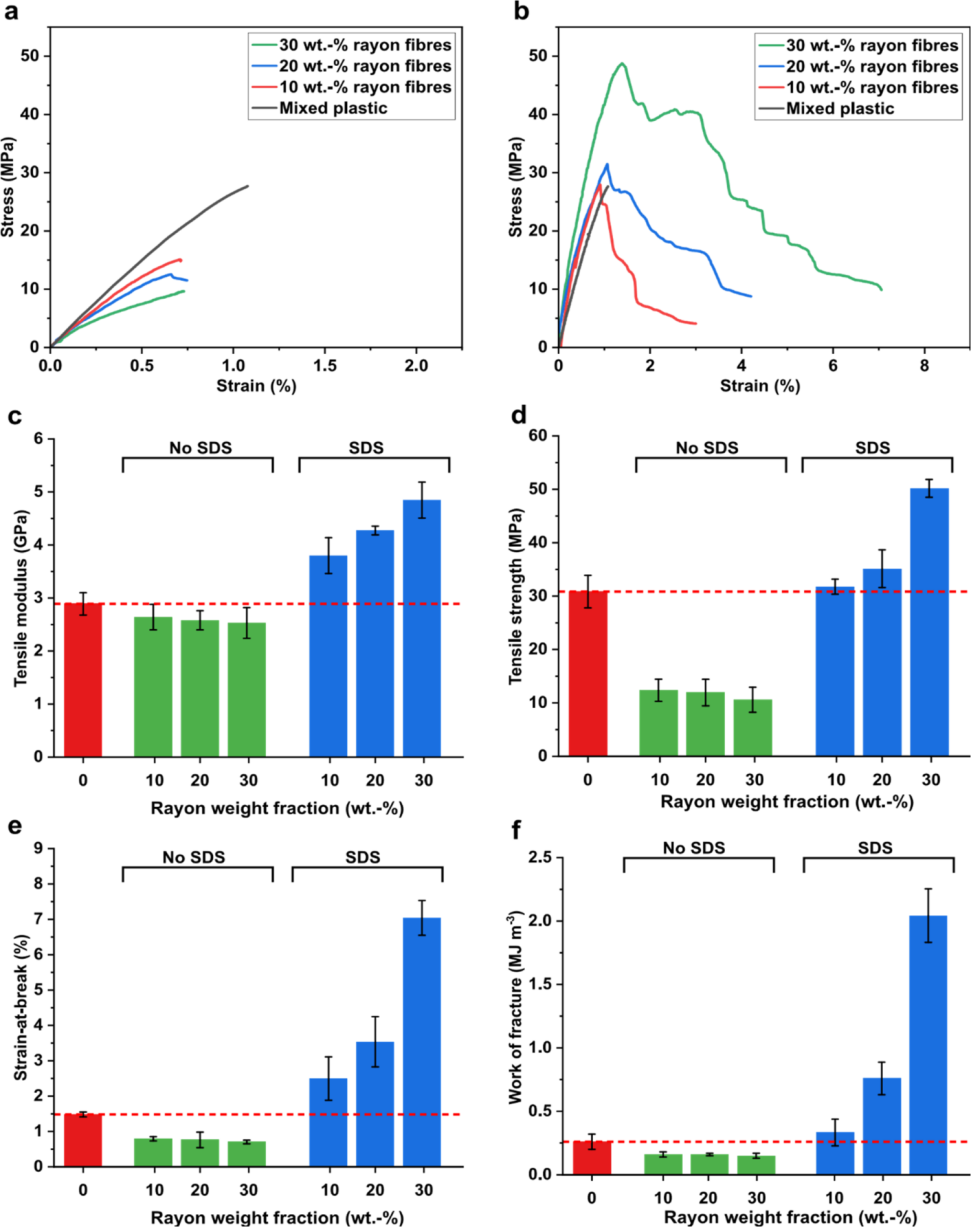
Representative tensile stress–strain curves of unreinforced, 10, 20, and 30 wt % rayon fiber-reinforced shredder residue mixed plastic (a) without SDS and (b) with SDS as the processing aid. (c) Tensile modulus, (d) tensile strength, (e) strain-at-break, and (f) work of fracture of unreinforced, 10, 20, and 30 wt % rayon fiber-reinforced shredder residue mixed plastic without SDS and with SDS as the processing aid.

Figure 5a,b shows the representative uniaxial tensile stress–strain curves of unreinforced and rayon fiber-reinforced shredder residue mixed plastic composites produced without and with SDS as the processing aid during composite processing, respectively. After the initial linear elastic response, melt-consolidated shredder residue mixed plastic fractured catastrophically (see the black line in Figure 5a,b), characterized by a sharp decrease in load-bearing capacity to zero after peak load was reached. Such brittle behavior can be attributed to the immiscibility of ABS, PS, PP, PE, and other unknown polymers at a molecular level.^18,55,56^ We have previously^20^ discussed the immiscibility of these specific four polymers based on their solubility parameters, should the readers be interested. Immiscibility at the molecular level leads to phase separation within the blend, where distinct polymer domains form, each with its own mechanical response. This results in localized regions of high stress at the interfaces between these domains, leading to premature failure when subjected to mechanical loading.

It can also be seen from Figure 5a that the introduction of rayon fibers into the shredder residue mixed plastic matrix without using SDS as a processing aid not only resulted in a brittle composite that fractures catastrophically but also possessed poorer mechanical performance than unreinforced shredder residue mixed plastic (see Figure 5c–e, green columns). The tensile modulus, tensile strength, and tensile strain-at-break of the 30 wt % rayon fiber-reinforced shredder residue mixed plastic composites were found to be ∼2.5 GPa, ∼11 MPa, and 0.7%, respectively. By comparison, unreinforced shredder residue mixed plastic (Figure 5c–e, red columns) possesses a higher tensile modulus of 2.9 GPa, tensile strength of 31 MPa, and tensile strain-at-break of 1.5%. This is due to the presence of a laminated composite architecture and a high porosity as a result of poor impregnation of the shredder residue mixed plastic matrix into the rayon fiber network (see Figure 4b and Table 1). The dry rayon fiber network is held together only by low frictional force and fiber entanglement. Therefore, upon fracture of the polymer matrix, the rayon fibers do not contribute to the overall load-bearing capacity of the composite.

Model rayon fiber-reinforced shredder residue mixed plastic composites processed using SDS as a processing aid were found to possess different uniaxial tensile responses. These composites exhibited a progressive failure (see Figure 5b). Comparing the stress–strain curves between the rayon fiber-reinforced shredder residue mixed plastic composites processed with and without SDS, it can be seen that the elastic region of both types of composites failed at a similar strain. This is indicative that the addition of rayon fibers did not delay the onset fracturing point of the brittle shredder residue mixed plastic matrix. Instead, the observed pseudoductility in Figure 5b was provided by the composite construct. The tensile modulus, tensile strength, and strain-at-break of rayon fiber-reinforced shredder residue mixed plastic composite produced with SDS as a processing aid were found to be 3.8 GPa, 32 MPa, and 2.5%, respectively, at 10 wt % fiber loading.

A further increase in loading fraction of rayon fibers to 30 wt % increased the tensile modulus, tensile strength, and strain-at-break to 4.8 GPa, 50 MPa, and 7%, respectively. It is expected that this improvement in tensile modulus, strength, and strain-at-break translates into enhanced work of fracture (calculated from the area under the stress–strain curves, Figure 5f). When SDS was used with high rayon fiber loading, the work of fracture increased to 2 MJ m^–3^, a 700% increase compared to unreinforced shredder residue mixed plastic. Similar observation in terms of improved ductility and toughness with regenerated cellulose reinforcement were also obtained by Bax and Müssig^32^ for randomly oriented short Cordenka fiber-reinforced PLA composites, Graupner and Müssig^33^ for randomly oriented short Lyocell fiber-reinforced polypropylene (PP) and PLA composites, as well as Shamsuddin et al.^21^ for continuous Cordenka fiber-reinforced polyhydoxybutyrate (PHB) composites.

### 3.4 Tensile Fracture Surface of Model Rayon Fiber-Reinforced Shredder Residue Mixed Plastic Composites

**Figure 6:**
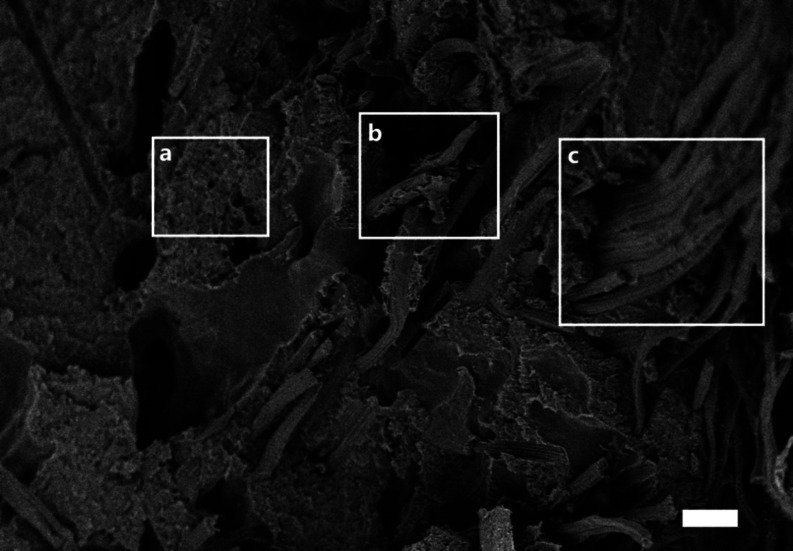
Exemplary tensile fracture surface of 30 wt % rayon fiber composite processed using SDS as a processing aid showing (a) a brittle fracture, (b) single fiber pulling out, and (c) fiber bundle pulling out. Scale bar = 40 μm.

The tensile fracture surface of 30 wt % rayon fiber composites fabricated with SDS as a processing aid was further studied to investigate the origin of the observed progressive failure in their mechanical response. The feature of a brittle fracture can be seen in this sample (Figure 6, inset a), which is characterized by a rough and jagged appearance with little polymer matrix deformation. This is due to cracks propagating rapidly through the polymer matrix, a consequence of its brittleness, creating such an uneven surface. The appearance of this fracture surface corroborates with the lack of delay in the onset fracturing point, as previously discussed. Single rayon fiber pullout (Figure 6, inset b) and the pulling out of rayon fiber bundles (Figure 6, inset c) can also be observed. Typically, the agglomeration or bundling of fibers in a polymer matrix is often considered as a defect.^57^ Nevertheless, it was recently suggested that such agglomeration/bundling may enhance fracture toughness as higher energy is required for cracks to propagate around these fiber bundles to cause them to pull out.^58^ This is because when a crack encounters a fiber bundle, the local fracture toughness increases, slowing down the propagation of the crack.^59^ The crack then diverts around the fiber bundle (if the fibers do not fracture), causing the energy release rate to exceed the fracture toughness of the surrounding matrix, resulting in unstable crack jumps. During these crack jumps, the crack rapidly propagates via the stress-concentrated fiber bundle/polymer matrix interface until it encounters another fiber bundle, slowing the crack propagation once more.^60^ Such an effect leads to a stick–slip pattern in the stress–strain curve, which is observed in Figure 5b after peak stress was reached.

### 3.5 Charpy Impact Strength of Model Rayon Fiber-Reinforced Shredder Residue Mixed Plastic Composites

**Figure 7:**
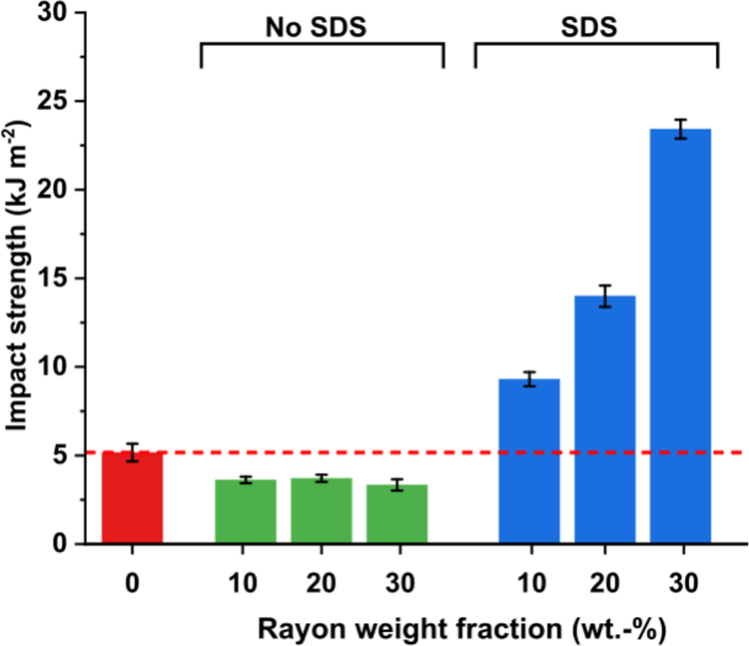
Charpy impact strength of unreinforced, 10, 20, and 30 wt % rayon fiber-reinforced shredder residue mixed plastic.

The impact strength of a material is one of the crucial properties for assessing its ability to absorb and withstand sudden impact events, which are common occurrences in real-world applications. This property serves as a fundamental determinant of material durability, thereby suggesting its suitability and use across diverse industrial sectors. Figure 7 presents the flatwise Charpy impact strength of unreinforced and rayon fiber-reinforced shredder residue mixed plastic composites. As expected, due to the brittleness of the unreinforced shredder residue mixed plastic, it possesses a low impact strength of 5 kJ m^–2^. Despite PS being one of the most brittle polymers with an impact strength of 12 kJ m^–2^,^61,62^ the shredder residue mixed plastic displays notably lower impact strength. Without SDS as a processing aid, the Charpy impact strength of rayon fiber-reinforced shredder residue mixed plastic composites is worse than that of unreinforced shredder residue mixed plastic. At 10 wt % rayon fiber loading, the resulting composite possessed an impact strength of only 3.6 kJ m^–2^. A further increase in rayon fiber loading to 30 wt % decreased the impact strength further to 3.3 kJ m^–2,^ a 35% reduction from unreinforced shredder residue mixed plastic. As previously shown in Figure 4, the lack of impregnation of the rayon fiber network implies that load cannot be effectively transferred to the more ductile fibers. The dry rayon fiber network behaves as a flaw that ultimately decreased the impact strength of the rayon fiber-reinforced shredder residue mixed plastic composites. The better impregnated rayon fiber-reinforced shredder residue mixed plastic composites produced with SDS as a processing aid was found to significantly improve the impact strength (see Figure 7, blue columns). The Charpy impact strength of the composite increased by 80% to 9 kJ m^–2^ at 10 wt % rayon fiber loading. At 20 and 30 wt % rayon fibers, the resulting rayon fiber-reinforced shredder residue mixed plastic composites possessed an impact strength of 14 and 24 kJ m^–2,^ an increase of 170 and 353% from unreinforced shredder residue mixed plastic. These values are comparable to polycarbonate^63^ and plexiglas.^64^ This stems from the energy dissipating pullout of the more ductile rayon fiber bundles from the shredder residue mixed plastic matrix.

## 4 Conclusions

This work demonstrated that low-value shredder residue mixed plastics arising from WEEE and ELV that are destined for landfill or incineration can be upcycled into higher-value composite materials. An immiscible polymer blend consisting of shredder residue mixed plastics is brittle, with a tensile modulus of 2.9 GPa, a tensile strength of 30 MPa, a strain-at-failure of 1.5%, a work of fracture of 0.25 MJ m^–3^, and a Charpy impact strength of 5 kJ m^–2^. By reinforcing the brittle shredder residue mixed plastic matrix with ductile regenerated viscose rayon fibers, pseudoductile rayon fiber-reinforced shredder residue mixed plastic composites can be produced. This can be attributed to the introduction of additional energy dissipating mechanisms associated with the pullout of the rayon fiber (bundles), as confirmed by the appearance of a stick–slip pattern in the composite’s stress–strain curves. At 30 wt % rayon fiber loading, the resulting composite possessed a tensile modulus of 5 GPa, a tensile strength of 50 MPa, a strain-at-failure of 7%, a work of fracture of 2 MJ m^–3^, and a Charpy impact strength of 25 kJ m^–2^. In this work, the importance of processing on the final properties of the resulting rayon fiber-reinforced shredder residue mixed plastic composites was highlighted. The aforementioned best-performing composite properties were processed in the presence of a surfactant to ensure the rayon fibers can be dispersed into the mixed plastic matrix. Without a surfactant as a processing aid, rayon fiber-reinforced mixed plastic composites possessed a laminated construct and poor matrix impregnation was obtained. At the same 30 wt % rayon fiber loading, the resulting poorly impregnated rayon fiber composites possessed only a tensile modulus of 2.5 GPa, a tensile strength of 10 MPa, a strain-at-failure of 0.7%, a work of fracture of 0.1 MJ m^–3^, and a Charpy impact strength of 2.5 kJ m^–2^.

## Supporting Information Available

It provides the particle size distribution of shredder residue mixed plastic powder used in this work and the microscopy images of the model rayon fiber-reinforced shredder residue mixed plastics (PDF)
