# Supplementary material for: Upgrading Mixed Plastic Waste through Industrial Symbiosis: Pseudoductile Regenerated Cellulose Fiber-Reinforced Shredder Residue Composites
Source: ACS Appl Polym Mater. 2024 Nov 18;6(23):14598–607. doi: 10.1021/acsapm.4c02728 (PMC11650560; doi:10.1021/acsapm.4c02728)
Supplement: Supplementary file 1 — ap4c02728_si_001.pdf [file ap4c02728_si_001.pdf]

Supplementary information

Upgrading mixed plastic waste through industrial symbiosis: Pseudo-ductile regenerated cellulose fibre-reinforced shredder residue composites

Kanjanawadee Singkronart[#], Jiayi Amy Sun[§], Siti Ros Shamsuddin[§], Koon-Yang Lee[§†]*

#National Metal and Materials Technology Centre, National Science and Technology Development Agency, 10210, Pathum Thani, Thailand

§Department of Aeronautics, Imperial College London, South Kensington Campus, SW7 2AZ, London, United Kingdom

†Institute for Molecular Science and Engineering (IMSE), Imperial College London, SW7 2AZ, London, United Kingdom

*Corresponding author. Email: koonyang.lee@imperial.ac.uk

Table of content

Figure S1. Particle size distribution of shredder residue mixed plastic powder used in this work…………………………………………………………………………………….....S2

Figure S2. Microscope images of model rayon fibre-reinforced shredder residue mixed plastic consisting of (a) 10 wt.-% rayon fibres processed with SDS, (b) 10 wt.-% rayon fibres processed without SDS, (c) 20 wt.-% rayon fibres processed with SDS and (d) 20 wt.-% rayon fibres processed without SDS. Scale bar = 2 mm……………………………………………………….S3

Figure S3. Microscope images of 30 wt.-% model rayon fibre-reinforced shredder residue mixed plastic processed without SDS as a processing aid, showing the lack of mixed plastic matrix impregnation. It can be seen that this particular sample can be (a) easily opened in mode I opening

and (b) closer inspection of the construct shows a lack of matrix impregnation. Scale bar = 2 mm.

…………………………………………………………………………………………………...S4

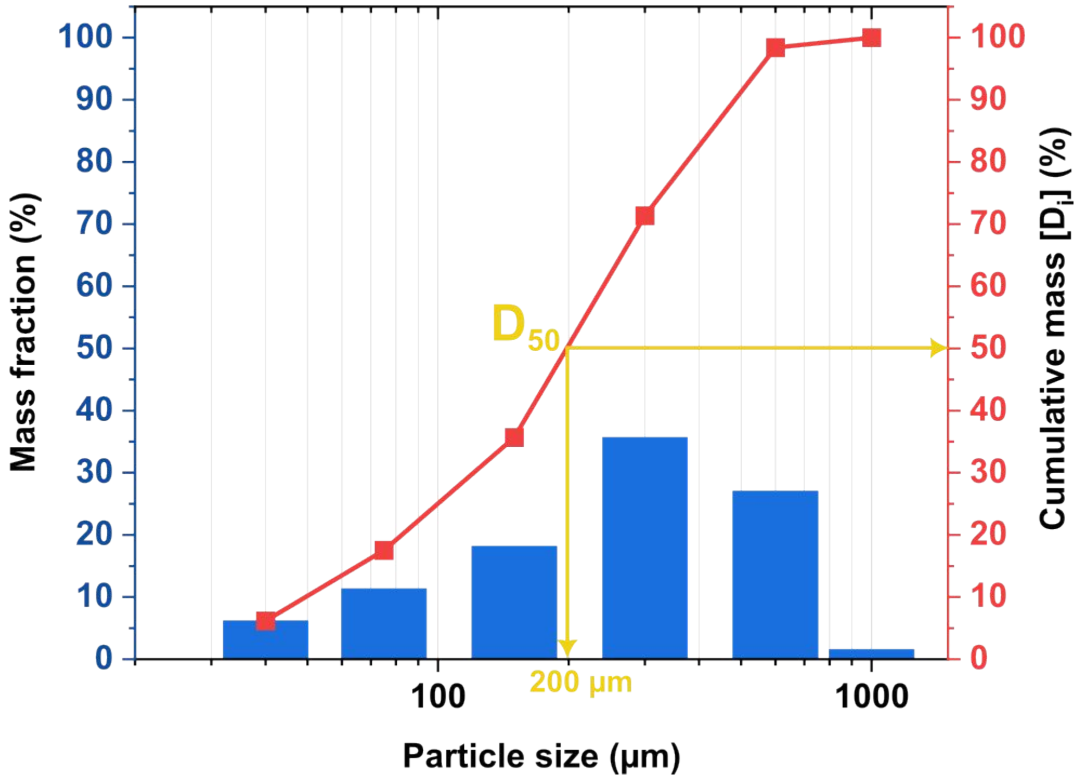

Figure S1. Particle size distribution of shredder residue mixed plastic powder used in this work.

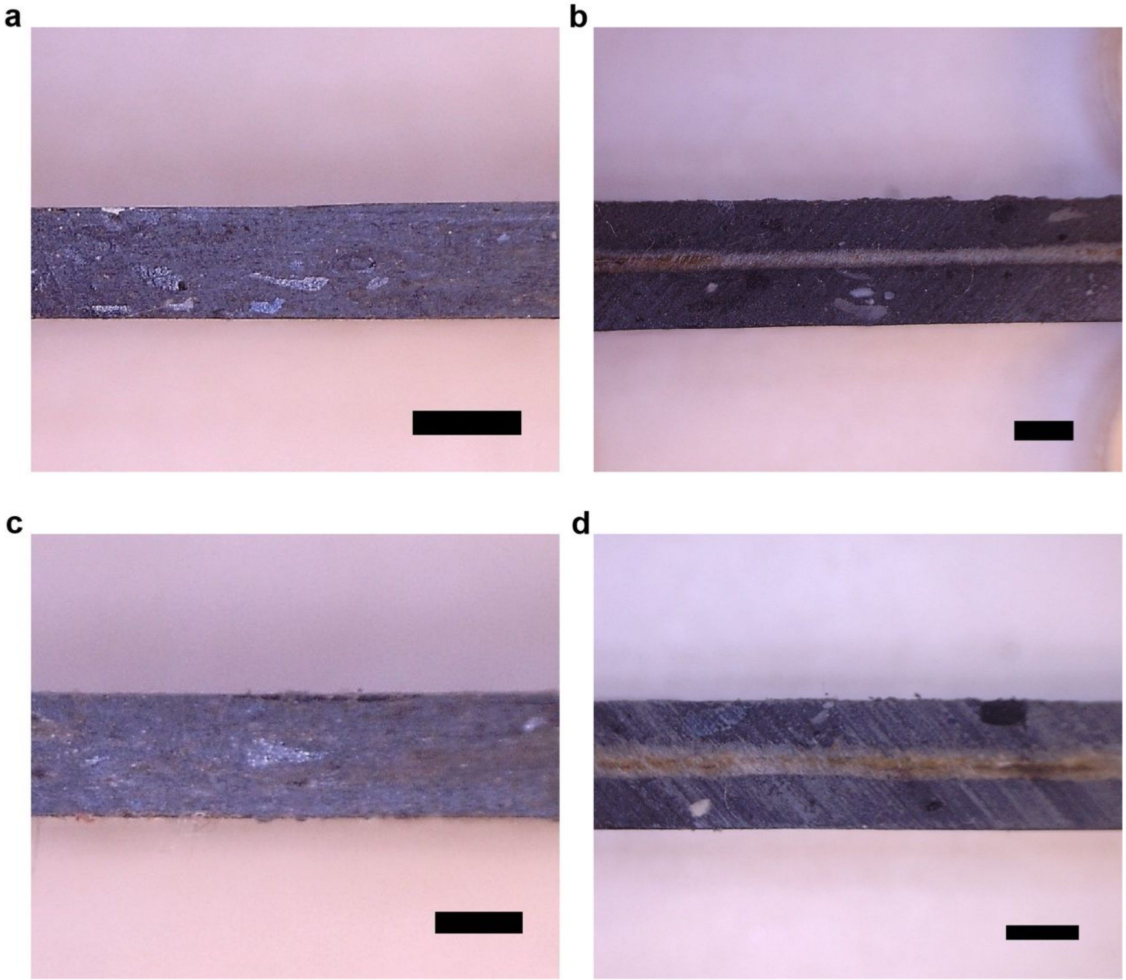

Figure S2. Microscope images of model rayon fibre-reinforced shredder residue mixed plastic consisting of (a) 10 wt.-% rayon fibres processed with SDS, (b) 10 wt.-% rayon fibres processed without SDS, (c) 20 wt.-% rayon fibres processed with SDS and (d) 20 wt.-% rayon fibres processed without SDS. Scale bar = 2 mm.

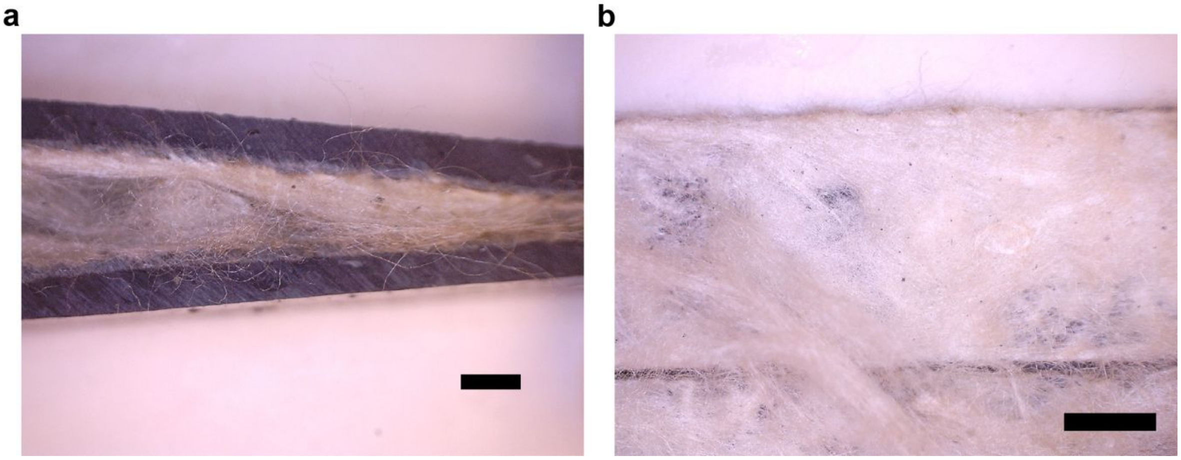

Figure S3. Microscope images of 30 wt.-% model rayon fibre-reinforced shredder residue mixed plastic processed without SDS as a processing aid, showing the lack of mixed plastic matrix impregnation. It can be seen that this particular sample can be (a) easily opened in mode I opening and (b) closer inspection of the construct shows a lack of matrix impregnation. Scale bar = 2 mm.
